# Distinct differences in immunological properties of equine orthobiologics revealed by functional and transcriptomic analysis using an activated macrophage readout system

**DOI:** 10.3389/fvets.2023.1109473

**Published:** 2023-02-16

**Authors:** Lynn M. Pezzanite, Lyndah Chow, Gregg M. Griffenhagen, Luke Bass, Laurie R. Goodrich, Renata Impastato, Steven Dow

**Affiliations:** ^1^Department of Clinical Sciences, College of Veterinary Medicine and Biomedical Sciences, Colorado State University, Fort Collins, CO, United States; ^2^Department of Microbiology, Immunology and Pathology, College of Veterinary Medicine and Biomedical Sciences, Colorado State University, Fort Collins, CO, United States

**Keywords:** biologic, intra-articular, mesenchymal stromal cell, autologous conditioned serum, equine, platelet rich plasma

## Abstract

**Introduction:**

Multiple biological therapies for orthopedic injuries are marketed to veterinarians, despite a lack of rigorous comparative biological activity data to guide informed decisions in selecting a most effective compound. Therefore, the goal of this study was to use relevant bioassay systems to directly compare the anti-inflammatory and immunomodulatory activity of three commonly used orthobiological therapies (OTs): mesenchymal stromal cells (MSC), autologous conditioned serum (ACS), and platelet rich plasma (PRP).

**Methods:**

Equine monocyte-derived macrophages were used as the readout system to compare therapies, including cytokine production and transcriptomic responses. Macrophages were stimulated with IL-1ß and treated 24 h with OTs, washed and cultured an additional 24 h to generate supernatants. Secreted cytokines were measured by multiplex immunoassay and ELISA. To assess global transcriptomic responses to treatments, RNA was extracted from macrophages and subjected to full RNA sequencing, using an Illumina-based platform. Data analysis included comparison of differentially expressed genes and pathway analysis in treated vs. untreated macrophages.

**Results:**

All treatments reduced production of IL-1ß by macrophages. Secretion of IL-10 was highest in MSC-CM treated macrophages, while PRP lysate and ACS resulted in greater downregulation of IL-6 and IP-10. Transcriptomic analysis revealed that ACS triggered multiple inflammatory response pathways in macrophages based on GSEA, while MSC generated significant downregulation of inflammatory pathways, and PRP lysate induced a mixed immune response profile. Key downregulated genes in MSC-treated cultures included type 1 and type 2 interferon response, TNF-α and IL-6. PRP lysate cultures demonstrated downregulation of inflammation-related genes IL-1RA, SLAMF9, ENSECAG00000022247 but concurrent upregulation of TNF-α, IL-2 signaling, and Myc targets. ACS induced upregulation of inflammatory IL-2 signaling, TNFα and KRAS signaling and hypoxia, but downregulation of MTOR signaling and type 1 interferon signaling.

**Discussion:**

These findings, representing the first comprehensive look at immune response pathways for popular equine OTs, reveal distinct differences between therapies. These studies address a critical gap in our understanding of the relative immunomodulatory properties of regenerative therapies commonly used in equine practice to treat musculoskeletal disease and will serve as a platform from which further *in vivo* comparisons may build.

## Introduction

Osteoarthritis (OA) represents one of the most common conditions treated by equine practitioners and is estimated to affect 80% of horses over 15 years of age and up to 2/3 of Thoroughbred racehorses (1–3). Despite this high prevalence, no approved pharmacological intervention, biological therapy, or procedure prevents or reverses progressive destruction of the degenerative joint. Orthobiologic therapies (OTs) are increasingly popular but their true efficacy remains controversial due to lack of rigor in clinical study design and the lack of demonstrated consistency in product formulation. Progressive joint degeneration is increasingly thought to be a multifactorial disease in which the innate immune system, particularly macrophages, plays an important role in regulating and perpetuating low-grade inflammation, resulting in continued articular cartilage breakdown for years following initial joint trauma. Synovial macrophages are the most numerous immune cells in the joint and among the most immunologically active cells, responding to signals released from cartilage degradation products, among other environmental triggers (4). Macrophages display high phenotypic and functional heterogeneity ranging from classical pro-inflammatory (M1) macrophages to reparative (M2) macrophages (5, 6). Alterations in synovial macrophage functional activity have been implicated in the pathogenesis of OA, propagating cartilage destruction and synovitis, with a higher ratio of M1/M2 associated with greater severity in human knee OA (7–14). Therefore, efforts to reduce inflammation associated with progression of OA would include resident synovial macrophages as well as infiltrating inflammatory monocytes as primary targets for immune modulation.

Orthobiologic therapies (OTs) frequently used in veterinary practice to treat OA include autologous conditioned serum (ACS) (15, 16), platelet rich plasma (PRP) (17–21), and mesenchymal stromal cells (MSC) (22, 23). However, relatively little work has been done to evaluate and compare the biological activities of these compounds more fully (24–27). There is also a paucity of evidence to support optimal processing and storage conditions, currently recommended doses, and evidence-based protocols for application of OTs clinically (15, 16, 28, 29). The decision on which OT to use in specific disease conditions (e.g., soft tissue vs. cartilage injury) is often based on incomplete information on the specific pathological physiology and thus may lead to inappropriate choices regarding the most effective OT.

Therefore, the purpose of the current study was to directly evaluate and compare the immune modulatory properties of three commonly used OTs using multiple functional and transcriptomic readouts. This in-depth analysis provides a more complete understanding of the different OT mechanisms of action and how they may resemble and differ from one another. Specifically, the first aim was to determine the macrophage cytokine response to OTs using relevant cell culture assays with equine macrophages. The second aim was to use transcriptomic analysis of OT-treated macrophages to identify unique and potentially disease-modifying pathways and how they may differ between the three OTs. We hypothesized that all three OTs would suppress IL-1ß induced macrophage activation and would also activate unique and distinctive gene expression pathways in macrophages. This approach was based on the fact that IL-1ß is one of the key cytokines associated with cartilage degradation in OA, and a cytokine known to strongly activate synovial macrophages (30). The long-term goal of this study is to add to our understanding of the mechanisms by which OTs function to guide more evidence-based treatment decisions with different OT products.

## Methods

### Study overview

**Figure 1:**
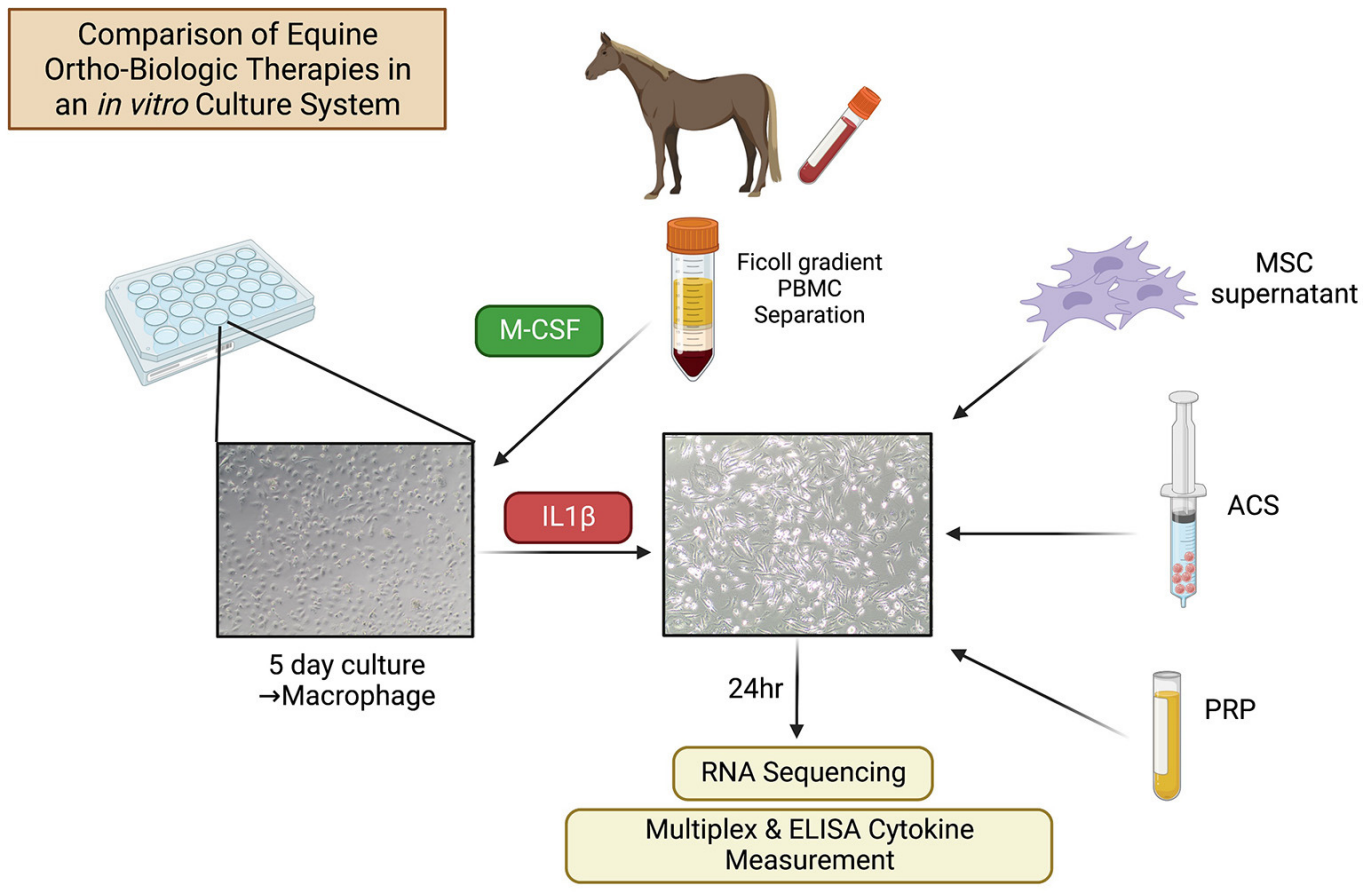
Schematic of study design. Equine macrophages were generated from PBMC by adherence of the monocytes, followed by treatment with M-CSF. After 5 days in culture, macrophages were stimulated with IL-1ß (10 ng/ml) and orthobiological therapies (MSC-CM, PRP, ACS) from *n* = 3 donor horses were added to macrophage cultures at 1:3 cell ratio. After 24 h of treatment, macrophages were washed with PBS and cultured in complete media an additional 24 h, at which time supernatants were collected for cytokine analysis and macrophage cells were collected for RNA sequencing.

Three healthy Quarter Horses (aged 2, 5, and 5 years; one gelding and two mares) were used as donors of blood and bone marrow aspirate to prepare orthobiologic therapies. Two additional Quarter Horse geldings, aged 3 and 5 years, were used as blood donors to generate monocyte-derived macrophage cultures. All procedures were approved by the University's Institutional Animal Care and Use Committee (IACUC protocol #927) and were performed in accordance with CONSORT guidelines according to national guidelines under which the university operates and NIH guidelines for the Care and Use of Laboratory Animals (8th edition). Study overview is summarized in Figure 1.

### Orthobiologic therapy preparation

To isolate MSC, the sternum of donor horses (*n* = 3) was clipped and aseptically prepared. Bone marrow aspirate (5–15 ml) was obtained from the sternebrae using a jamshidi into a syringe containing 1 ml heparin (5,000 U/ml). Bone marrow aspirates were centrifuged by Ficoll density separation (Ficoll-Paque^TM^ Plus; GE Healthcare BioSciences) at 400 g for 18 mins to pellet red cells as previously described (31, 32). The mononuclear cell population was plated at 10,000 cells/cm^2^ and expanded in culture (37°C, 5% CO_2_, 95% humidity) to 80% confluence for approximately seven days in complete growth medium [Dulbecco's Modified Eagle's Medium (DMEM) with 1,000 mg/L glucose, 10% fetal bovine serum (FBS), penicillin (100 U/ml), streptomycin (100 μg/ml), 1M HEPES]. Cells were detached from flasks by trypsinization, then frozen at 5 × 10^6^ cells/ml in freeze media [90% FBS, 10% dimethyl sulfoxide (DMSO)] in liquid nitrogen vapor phase until further use. To generate MSC-CM for use in co-culture assays, cells were thawed quickly in a 37°C water bath and cultured 48 h in complete growth medium under standard incubation conditions (37°C with 5% CO_2_). MSC were subsequently plated at 100,000 cells/well on 24-well plates for 24 h and supernatants were collected and frozen at −80°C for use in immunoassays.

To prepare autologous conditioned serum (interleukin 1 receptor antagonist; IRAP), blood (60 ml) was drawn and incubated according to manufacturers' instructions (IRAP II, Arthrex, Naples, FL, USA 34108). Aliquots (1 ml) of IRAP were frozen at −80°C for later use in immunoassays. To prepare platelet rich plasma lysate, blood was drawn, processed according to manufacturers' instructions (Arthrex ACP Double Syringe System, Naples, FL, USA 34108), and frozen at −80°C in 1 ml aliquots for use in co-culture assays.

### Monocyte-derived macrophage cultures

To generate macrophage cultures, equine peripheral blood mononuclear cells were isolated from whole blood of two horses by density gradient centrifugation (Ficoll-Paque TM plus, GE Healthcare Bio-Sciences) and cultured in macrophage media (Dulbecco's Modified Eagle's Medium supplemented with 10% fetal bovine serum, non-essential amino acids, and penicillin/streptomycin antibiotics; SigmaAldrich) with human M-CSF (PeproTech, Rocky Hill, NJ USA 80553) at 30 ng/ml to stimulate differentiation into macrophages in 5 days, as previously described (33).

Equine monocyte-derived macrophages were stimulated with IL-1ß (10 ng/ml) and OTs (MSC-CM, PRP lysate, ACS) were added at the same time to macrophage culture media at a ratio of 1:3 OT to complete growth media in culture (i.e., 25% OT in culture media). The ratio of OT to growth media was determined in a pilot study titrating OT to growth media to determine the maximum volume at which OT could be added in culture media while still maintaining macrophage cell viability over 80% following 24 h in culture. Controls included IL-1ß stimulated and unstimulated macrophages. Following transient addition of treatments for 24 h in culture, macrophages were washed three times with phosphate buffered saline (PBS) and cultured an additional 24 h in macrophage culture media. At that time, culture supernatants were collected and assessed by multiplex bead assay (23 cytokines) and ELISA immunoassay (PGE-2, TGF-ß) to characterize the macrophage response. Macrophages were collected in RNA lysis buffer (350 μl/sample) and frozen at −20°C until RNA isolation was performed.

### ELISA for cytokine and PGE quantification

An ELISA was used to measure the concentration of prostaglandin E2 (PGE-2 high sensitivity ELISA kit, Enzo Life Sciences, Inc. Farmingdale, NY 11735) and TGF-β (Human/Mouse/Rat/Porcine/Canine TGF-ß1 quantikine ELISA, R&D Systems, Inc. Minneapolis, MN 55413) in culture supernatants. Fluorescent bead-based multiplex assay (Milliplex MAP Equine Cytokine/Chemokine Magnetic Beads Multiplex Assay, Millipore Sigma, Burlington, MA, 01803) was used to quantify the concentrations of 23 analytes [Eotaxin/CCL11, FGF-2, Fractalkine/CS3CL1, G-CSF, GM-CSF, GRO, IFN, IL-1α, IL-1β, IL-2, IL-4, IL-5, IL-6, IL-8/CXCL8, IL-10, IL-12 (p70), IL-13, IL-17a, IL-18, IP-10, MCP-1, RANTES/CCL5 and TNFα] in cell culture supernatants.

### RNA sequencing

RNA was extracted from frozen samples using the RNeasy kit (Qiagen Germantown, MD) according to manufacturer's instructions and sent to Novogene Corporation Inc. (Sacramento, CA) for RNA sequencing. RNA quality was determined by bioanalyzer (Agilent Technologies, Santa Clara, CA) to have RIN (RNA integrity number) of over 9.0 for all samples. mRNA was enriched using oligo (dT) beads, followed by cDNA library generation using TruSeq RNA Library Prep Kit (Illumina, San Diego, CA). Sequencing was performed on Illumina Novaseq 6000 machine using 150 bp paired end reads.

### Data analysis

To analyze cytokine data, raw data was plotted and visually assessed for normality prior to statistical analysis. Cytokine data was then modeled individually using a linear mixed model [function *lmer* from the lme4 (34) and lmerTest (35) packages] with donor as a random effect to account for differences in donor cell lines. Each of the treatment groups (MSC-CM, ACS, PRP lysate, and negative control) were then compared individually with the positive control using estimated marginal means (package emmeans) (36) and Dunnett's test for multiple comparisons (37). When multiple treatments were found to be significantly different from the positive control, the comparisons were expanded to include evaluation of differences between all groups and Tukey's method for *p*-value adjustment was applied. For the cytokine secretion assays, orthobiologic treatment was modeled as the sole fixed effect. Statistical analyses, graphical analyses and graph generation were performed using Prism software v8.4.1 (GraphPad Software Inc., La Jolla, CA) and R version 4.1.2 “Bird Hippie” (R Foundation for Statistical Computing, Vienna, Austria) (38). For all analyses, statistical significance was assessed as *p* < 0.05.

To analyze RNA sequencing data, demultiplexed Fasq reads generated from Novogene were analyzed using Partek^®^ Flow^®^ software, v10.0 (Partek Inc. Chesterfield, MO). Reads were trimmed for Phred score of 20, adapters removed using cutadapt (39). Trimmed reads were aligned using STAR 2.7.3 using EquCab3.0 and annotated with Ensembl EquCab3.0.107. Feature counts were generated with HTseq (40). Differential analysis was computed using counts normalized to CPM, using DESeq (41). Pathway analysis was performed with GSEA (Gene Set Enrichment Analysis) v4.2.1using Hallmark pathways (42).

## Results

### Impact of OTs on cytokine and PGE secretion from IL-1ß activated macrophages

We first evaluated the impact of OTs on cytokine secretion by IL-1ß activated equine macrophages. This approach was based on the fact that IL-1ß is one of the key cytokines associated with cartilage degeneration in OA, and a cytokine known to strongly activate synovial macrophages (30). Given evidence that macrophages activated by inflammatory cytokines such as IL-1ß may be the key mediator cell for driving progressive OA, we modeled the impact of OTs on modulating cytokine production by activated equine macrophages, beginning with analysis of cytokine secretion.

**Figure 2:**
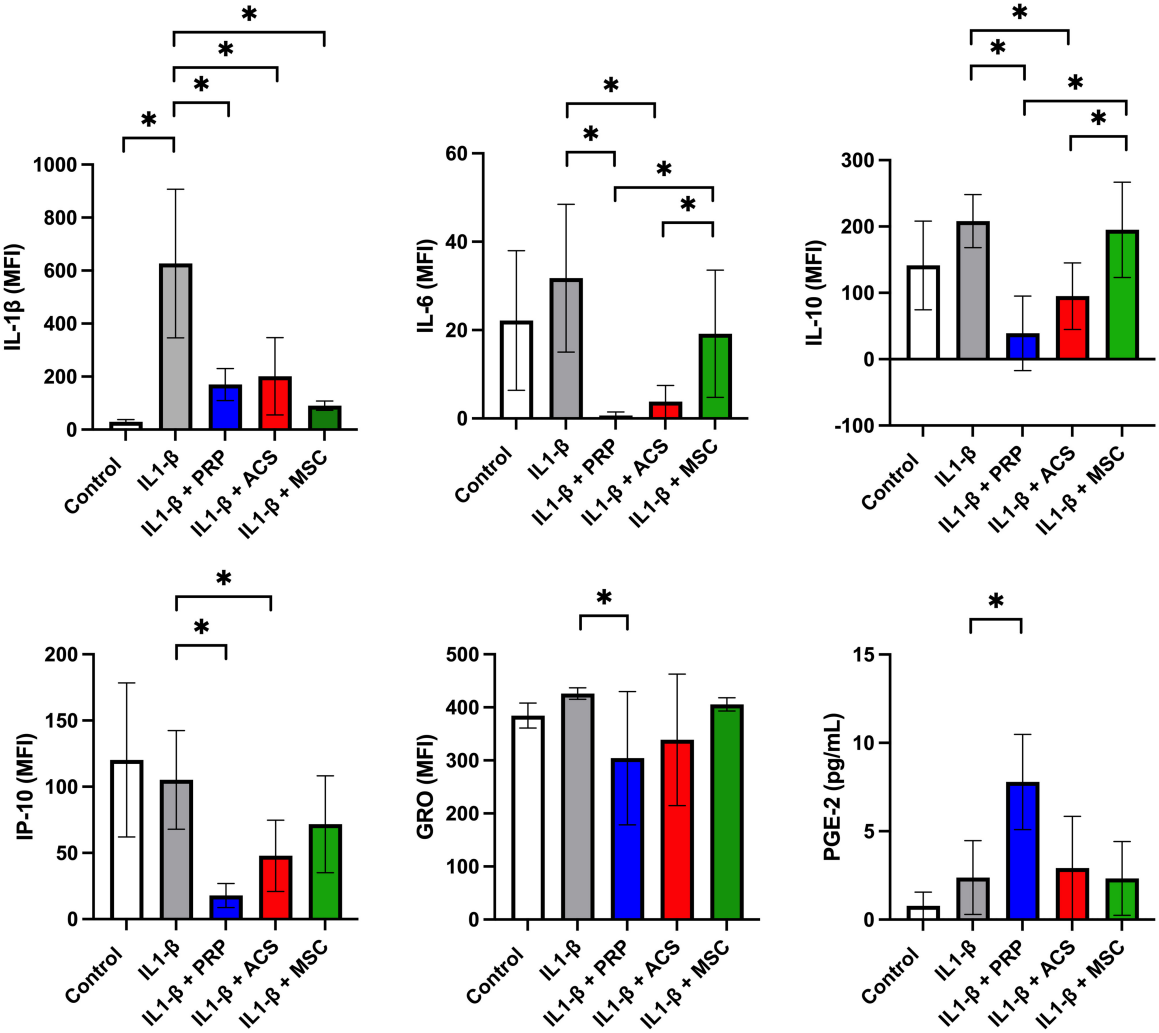
Effect of orthobiologic treatment on macrophage cytokine secretion. Orthobiologic therapies (platelet rich plasma or PRP, autologous conditioned serum or ACS, or mesenchymal stromal cell conditioned media or MSC) were added to culture media with equine monocyte derived macrophages and IL-1ß (10 ng/ml) for 24 h. Controls included unstimulated and IL-1ß stimulated macrophages. After 24 h, macrophages were washed in phosphate buffered saline and cultured an additional 24 h and culture supernatants collected and assessed for cytokine levels *via* multiplex immunoassay (23 cytokines) and ELISA immunoassay (PGE-2 and TGF-ß). Cytokine levels were measurable for ten cytokines *via* multiplex immunoassay (IL-1ß, IL-4, IL-6, IL-8, IFN-γ, IP10, GRO, IL-10, TNFα, and RANTES) and for one cytokine (PGE-2) *via* ELISA. Significant differences were seen between treatment groups for GRO, IL-1ß, IL-6, IL-10, IP-10, and PGE-2. There were no statistical differences noted for the remaining biomarkers assessed that achieved measurable levels by multiplex assay (IL-4, IL-8, IFN-γ, RANTES, and TNF-α). Levels of the remaining 14 cytokines assessed were below the detection limit of the multiplex assay (FGF-2, eotaxin, G-CSF, IL1α, GM-CSF, fractalkine, IL-13, IL-5, IL-18, IL-17A, IL-2, IL-12, and MCP-1) or ELISA (TGF-ß). Bars are mean and standard deviation of three biological replicates over two time points. *Statistical significance assessed at *p* < 0.05.

Cytokine concentrations were measurable for ten cytokines *via* multiplex immunoassay (IL-1ß, IL-4, IL-6, IL-8, IFN-γ, IP10, GRO, IL-10, TNFα, and RANTES) and for PGE-2 *via* ELISA (Supplementary material 1). Levels of the remaining 14 cytokines assessed were below the detection limit of the multiplex assay (FGF-2, eotaxin, G-CSF, IL1α, GM-CSF, fractalkine, IL-13, IL-5, IL-18, IL-17A, IL-2, IL-12, and MCP-1) or ELISA (TGF-ß). Significant differences were seen between treatment groups for IL-1ß, IL-6, IL-10, IP-10, GRO, and PGE-2 (Figure 2). Levels of IL-1ß were significantly higher in supernatants collected from IL-1ß treated macrophages compared to control media (*p* < 0.0001). All treatments reduced levels of IL-1ß as compared to the IL-1ß positive control with no significant differences between groups (MSC-CM *p* < 0.0001, PRP *p* < 0.0001, ACS *p* < 0.0001). Treatment with PRP lysate induced a significant reduction in GRO compared to IL-1ß treated control (*p* = 0.04) while inducing increasing levels of PGE-2 (*p* < 0.0001) with other treatments showing no significant changes in those specific cytokines. Treatment with PRP lysate and ACS resulted in lower levels of cytokines IL-6 (PRP *p* < 0.0001, ACS *p* < 0.0001), IL-10 (PRP *p* = 0.0002, ACS *p* = 0.015) and IP-10 (PRP *p* < 0.0001, ACS *p* = 0.005) relative to IL-1ß treated controls. The PRP lysate and ACS groups also had significantly lower levels of IL-6 (PRP lysate *p* = 0.007, ACS *p* = 0.03) and IL-10 (PRP lysate *p* = 0.0006, ACS *p* = 0.04) as compared to MSC-CM, while levels of IP-10 were not different between the ACS and MSC-CM groups. There were no statistical differences between orthobiologic treatment groups for the remaining biomarkers assessed that achieved measurable levels by multiplex assay (IL-4, IL-8, IFN-γ, RANTES, and TNF-α). Cytokine levels in unconditioned control media were below the detection limit of the multiplex assay.

### Transcriptomic analysis to understand the impact of OTs on activated macrophage immune pathways

**Figure 3:**
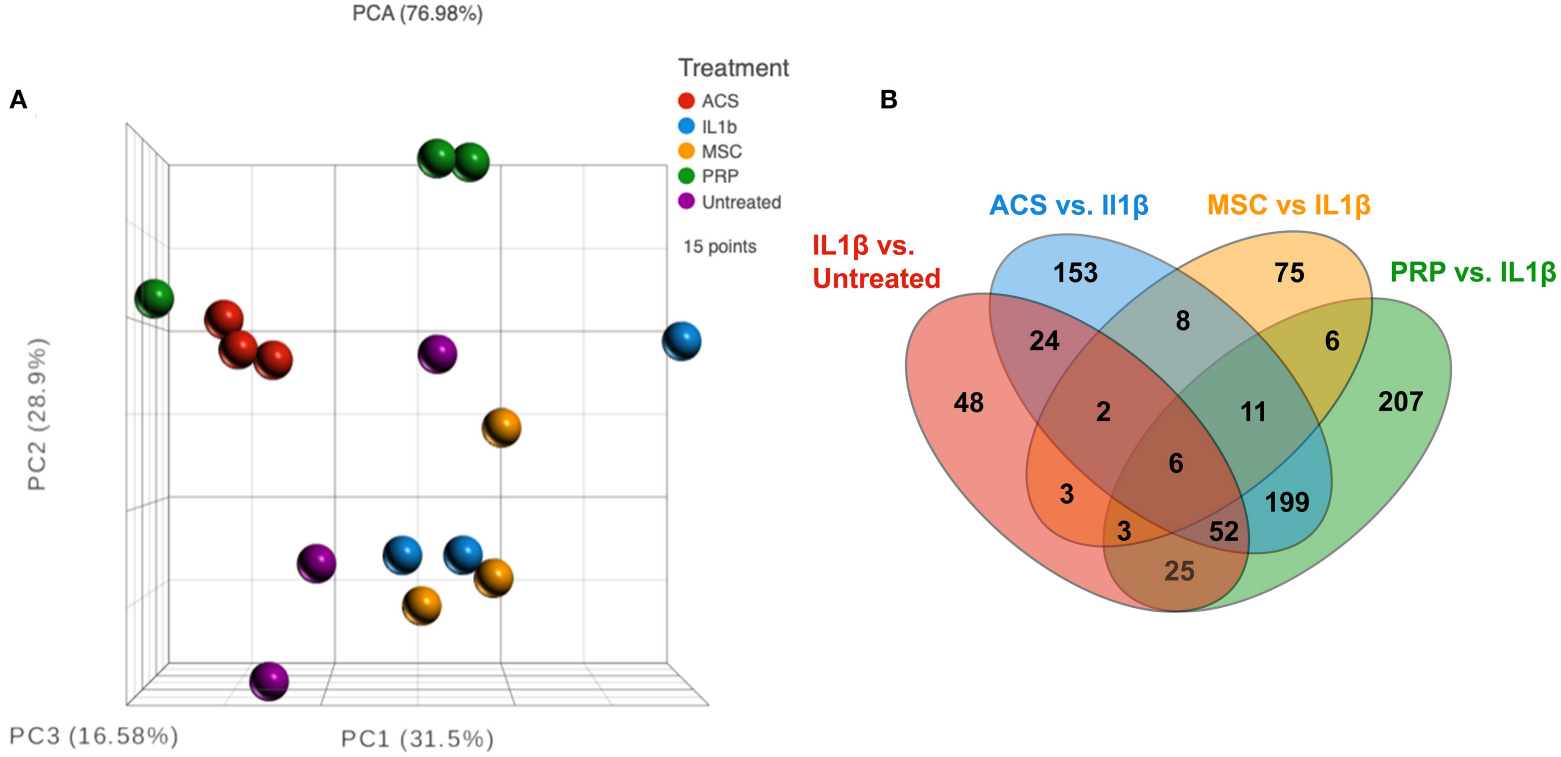
RNA sequencing analysis comparing three different types of treatment on macrophages. **(A)** Principle component analysis (PCA) or normalized counts from equine macrophages. Red dots show *n* = 3 technical replicate of macrophages treated with IL-1ß only, and purple show technical replicates of untreated macrophage. Other treatments include *n* = 3 biological replicates of orthobiologics including MSC supernatant (yellow), ACS (blue), and PRP (green), all treatments collected from *n* = 3 donor horses. **(B)** Venn diagram of differentially expressed genes from each comparison group, IL1-ß vs. untreated (red), IRAP vs. IL-1ß (blue), MSC vs. IL-1ß (yellow) and PRP vs. IL-1 ß (green). All comparisons except MSC were filtered by FDR adjusted *p* value of 0.05, MSC groups filtered using unadjusted *p* value of 0.05.

To further understand how OTs may modulate the function of IL-1ß activated macrophages, we next used RNA sequencing to interrogate the transcriptomic responses of macrophages exposed to three different OTs (Figure 3; Supplementary materials 2–4). Such an analysis can provide a much more comprehensive understanding of the impact of OT treatment on specific gene expression by macrophages, but more importantly on immune and other pathways that may be relevant to modulation of OA progression.

**Figure 4:**
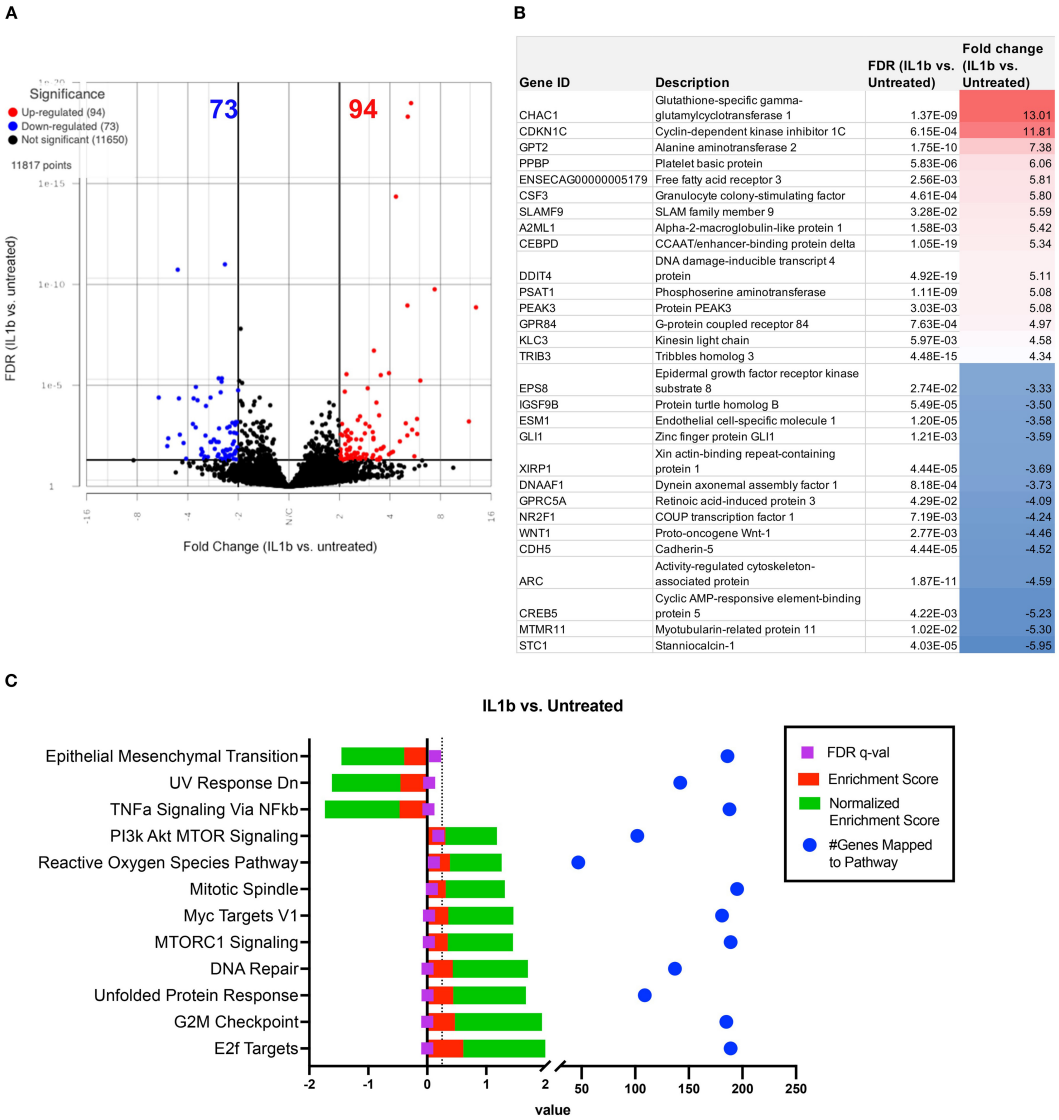
Differential gene expression and pathway analysis of IL-1ß stimulated macrophages compared to untreated. Differential gene expression and pathway analysis of IL-1ß stimulated macrophages compared to untreated. **(A)** Volcano plot of IL-1ß treatment vs. untreated. X axis shows fold change and y axis shows FDR adjusted *p*-value, with significantly upregulated genes shown in red dots and significantly downregulated genes in blue dots. Significance defined as FDR ≤ 0.05 fold change ≥2 or ≤ −2. **(B)** List of genes, description, FDR value and fold change of top 15 upregulated and downregulated genes in differential analysis results from IL-1ß vs. untreated samples. **(C)** GSEA (gene set enrichment analysis) results using normalized counts from *n* = 3 IL-1ß vs. untreated macrophages. FDR *p*-values shown in purple with dotted line on FDR = 0.25 for significance. Red bars show enrichment score (ES), green bars show Normalized enrichment score. Blue dots show total genes found in pathways. Pathways computed using hallmarks gene sets v2022.1.

As a first step in this analysis, we compared IL-1 activated macrophage transcriptomes to those of non-activated macrophages (Figures 3, 4). After the addition of IL-1β, RNA sequencing analysis indicated a visible separation from untreated macrophage samples by PCA (principal component analysis) (Figure 3A). Moderate changes in transcriptome were detected (Figure 4), including the upregulation of 94 genes with fold change ≥2 or ≤ -2 and significant FDR (false discovery rate) adjusted *p*-value of ≤ 0.05 (Figure 4A) and 73 significantly downregulated genes (Figure 3B). The most upregulated genes (Figure 4B) in IL-1ß activated macrophages included those related to inflammatory immune system process (SLAMF9 and PPBP), response to inflammatory stimuli (TRIB3, GPR84, and DDIT4), and metabolic process (CHAC1, PSAT1, PEAK3, and CEBPD). Downregulated genes mapped to categories including cell signaling (EPS8, GLI1, and WNT1) and to biological regulation of cellular construction, signaling, adhesion and differentiation (CDH5, CREB5, NR2F1, and STC1). Pathway analysis revealed that IL-1ß triggered significant downregulation of pathways involved in EMT transition, NFκβ signaling and upregulation of UV (DNA breakage) response, E2F targets, protein folding (structural) and G2M checkpoint (Figure 4C). Thus, the use of transcriptomic analysis provided important new and previously unpublished insights into how equine macrophages respond to activation by relevant joint inflammatory cytokines.

### Impact of OT treatment with MSC-CM on activated macrophage transcriptomes

**Figure 5:**
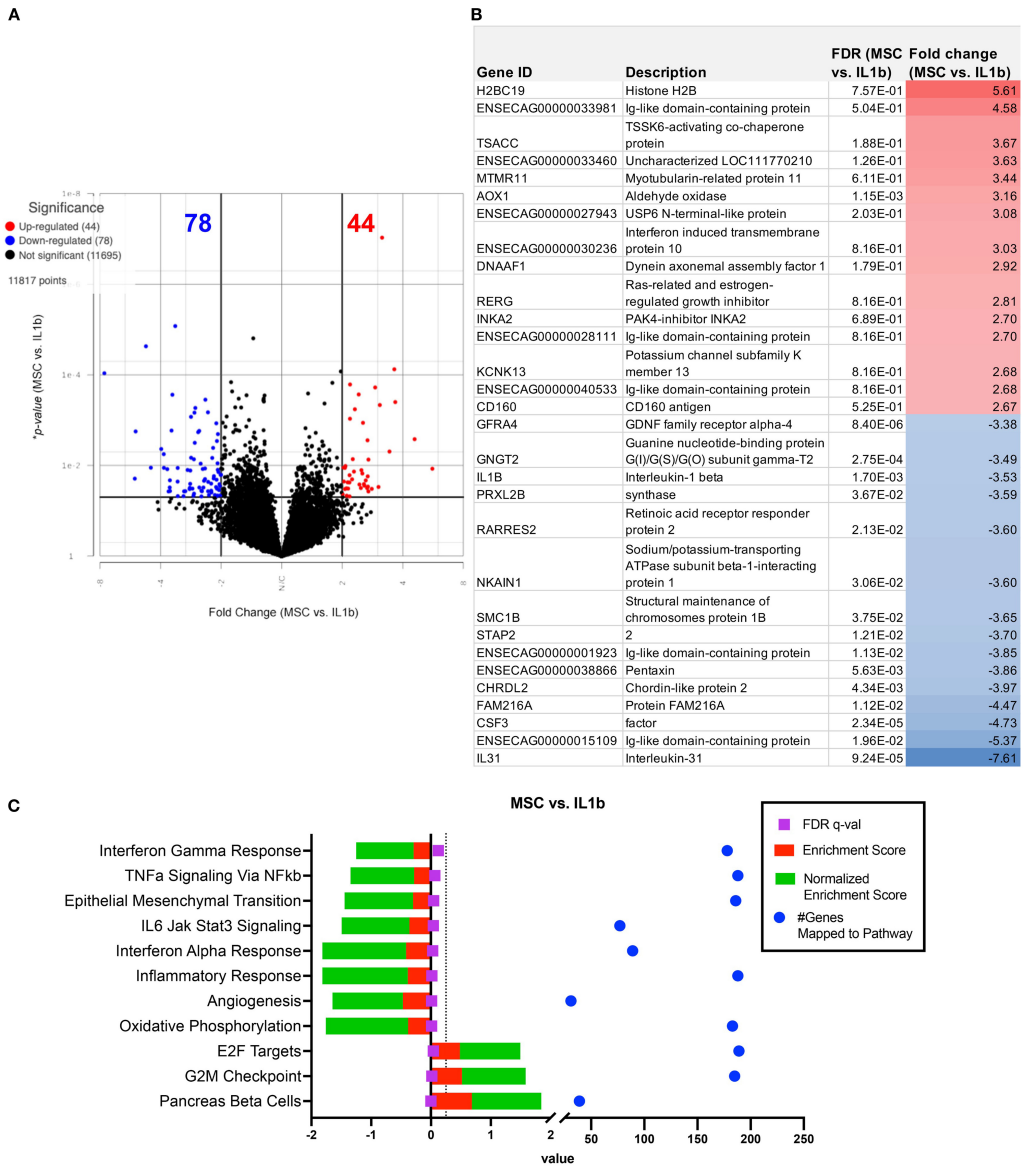
Differential gene expression and pathway analysis of MSC supernatant conditioned macrophages compared to IL-1ß stimulated. **(A)** Volcano plot of MSC treatment vs. IL-1ß. X-axis shows fold change and y-axis shows unadjusted adjusted *p*-value. Significance defined as *p*-value ≤ 0.05 fold change ≥2 or ≤ −2. **(B)** List of genes, description, *unadjusted *p*-value and fold change of top 15 upregulated and downregulated genes in differential analysis results from MSC supernatant treated vs. IL-1ß stimulated samples. **(C)** GSEA results using normalized counts from *n* = 3 MSC supernatant treated vs. IL-1ß stimulated macrophages. Legend shown in box to right.

We next conducted a series of studies to compare the impact of OT treatment on the activated macrophage transcriptome. The first OT evaluated was MSC, using CM from the MSC cultures to modulate IL-1ß activated macrophage immune responses. This analysis revealed that treatment of macrophages using supernatant collected from MSC cultures produced a smaller scale but anti-inflammatory change relative to that of the other OTs tested (Figures 3A, B). The PCA plot demonstrates MSC supernatant treated macrophages had 122 significantly differentiated genes relative to IL-1β conditioned macrophages (Figure 5A) with *p*-value (unadjusted) ≤ 0.05 and fold change ≥2. No significant genes were found when using FDR adjusted *p*-value. Out of these 122 significant DEGs, 75 were unique to the MSC-CM treatment (Figure 3B). The top 15 up or downregulated genes included biological processes such as immune system process (ENSECAG00000015109), stimulus response (IL-1ß, GNGT2), cytoskeletal reorganization, binding and signaling (IL-31, SMC1B, TSACC, RERG, INKA2) (Figure 5B). Overall, MSC-CM treatment caused a significant downregulation of inflammatory pathways such as type 1 and type 2 interferon response, TNF-α and IL-6 signaling (Figure 5C). This anti-inflammatory response can be seen in the downregulation of genes such as CXCL10, IL-1RN, IL-17 receptor, which are known as common mediators of chronic inflammation in OA joints (43).

### Macrophage transcriptomic response to treatment with PRP lysate

**Figure 6:**
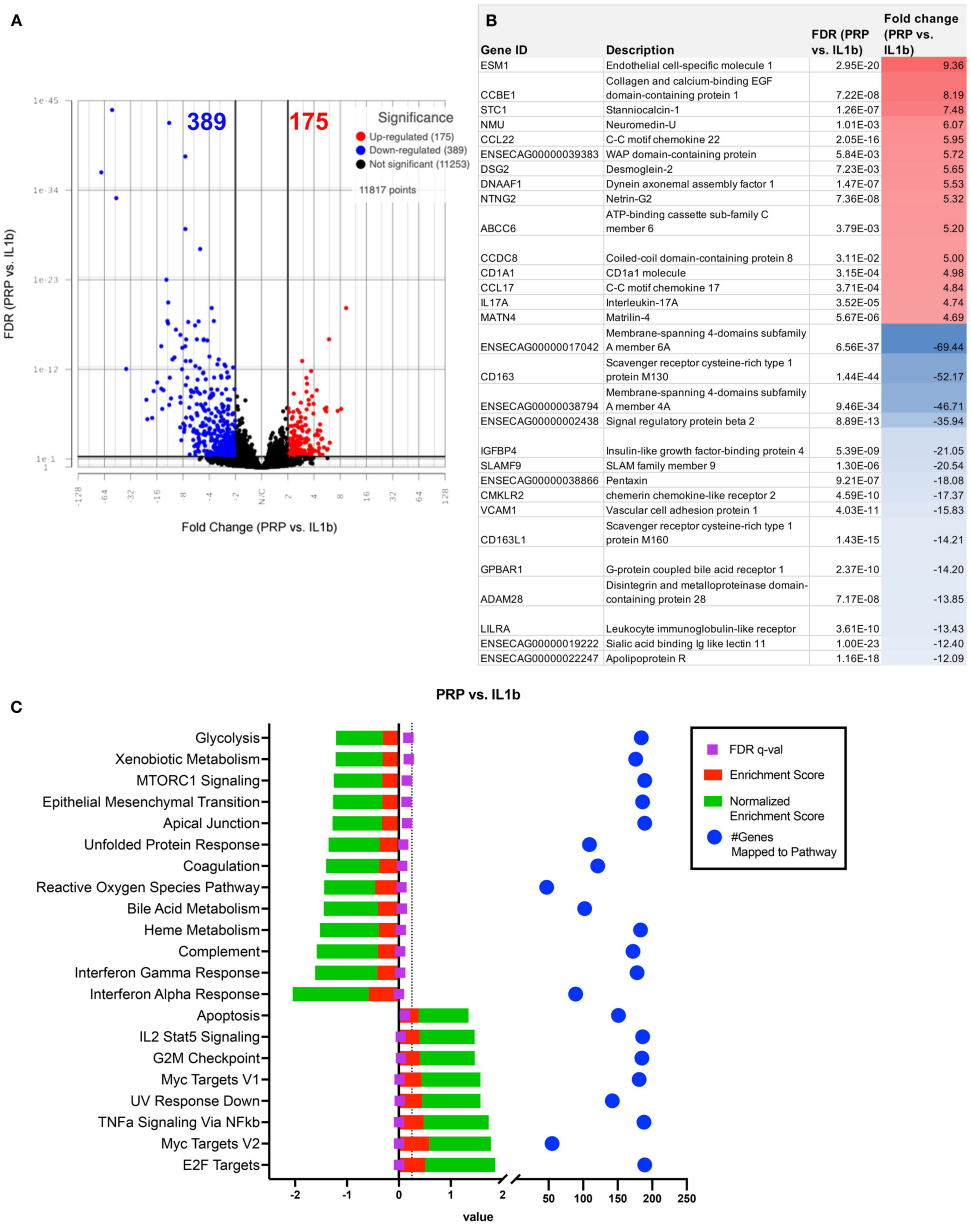
Differential gene expression and pathway analysis of PRP treated macrophages compared to IL-1ß stimulated. **(A)** Volcano plot of PRP treated vs. IL-1ß. X-axis shows fold change and y-axis shows unadjusted adjusted *p*-value. Significance defined as FDR adjusted *p*-value ≤ 0.05 fold change ≥2 or ≤ −2. **(B)** List of genes, description, FDR, and fold change of top 15 upregulated and downregulated genes in differential analysis results from PRP treated vs. IL-1ß stimulated samples. **(C)** GSEA results using normalized counts from *n* = 3 PRP treated vs. IL-1ß stimulated macrophages.

We next evaluated the impact of PRP lysate on activated macrophage transcriptomes. This analysis revealed that when compared to MSC-CM conditioned macrophages, PRP lysate treated cultures produced markedly larger changes in the transcriptome (Figure 3A), with 207 unique DEGs that were not found in the other treatment groups (Figure 3B). Differential analysis revealed a total of 564 significantly different (FDR ≤ 0.05 and fold change ≥ 2 or ≤ -2) genes (Figure 6A). Many of the downregulated genes in the PRP lysate treated group included inflammation related genes such as IL-1RA, SLAMF9, ENSECAG00000022247 (Figure 6B). These genes mapped to inflammatory pathways including type 1 and type 2 interferon signaling, complement and coagulation, as well as MTOR. All of which are molecular mechanisms implicated in the inflammatory response in OA pathogenesis (44). However, the PRP lysate treatment also generated upregulation of inflammatory response according pathway analysis, with significant upregulation of pathways such as TNF-α, IL-2 signaling, and Myc targets (Figure 6C). These pathways were not found to be significant in the IL-1β alone group and are therefore a unique reaction of the macrophages responding to the proteins contained in the PRP.

### Upregulation of multiple inflammatory pathways following treatment of activated macrophages with ACS

**Figure 7:**
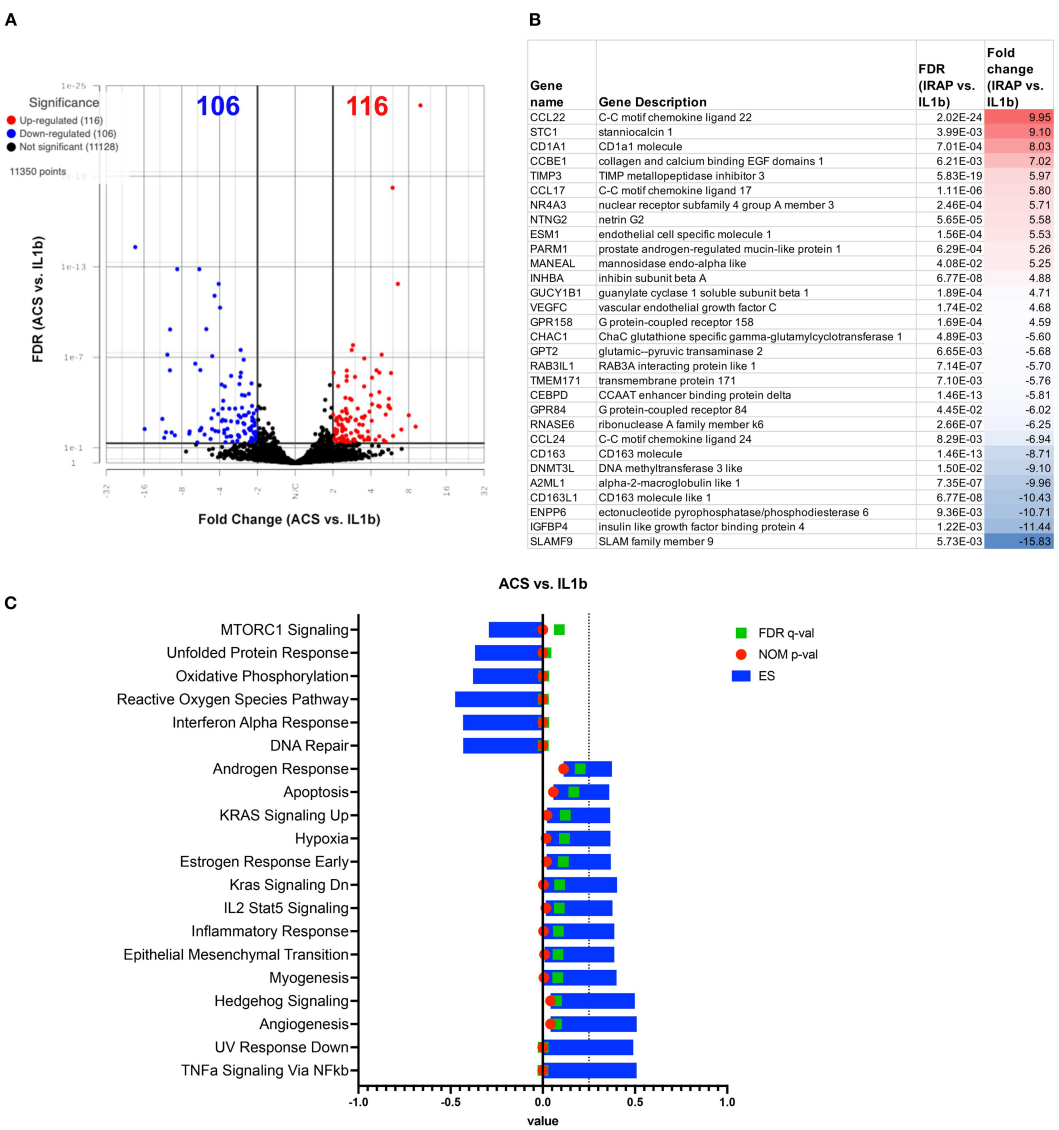
Differential gene expression and pathway analysis of ACS treated macrophages compared to IL-1ß stimulated. **(A)** Volcano plot of ACS treated vs. IL-1ß. X-axis shows fold change and y-axis shows unadjusted adjusted *p*-value. Significance defined as FDR adjusted *p*-value ≤ 0.05 fold change ≥2 or ≤ −2. **(B)** List of genes, description, FDR, and fold change of top 15 upregulated and downregulated genes in differential analysis results from ACS vs. IL-1ß stimulated samples. **(C)** GSEA results using normalized counts from *n* = 3 ACS treated vs. IL-1ß stimulated macrophages. Blue bars show ES, green FDR significant values, and nominal *p*-values shown in red.

We assessed the responses of IL-1ß activated macrophages to treatment with ACS. In contrast to the MSC-CM treatment, the ACS treatment induced a similar response to PRP lysate, creating a greater degree of change in the macrophage transcriptome, with a total of 564 significantly differentially expressed genes (FDR ≤ 0.05 and fold change ≥ 2 or ≤ -2) (Figure 7A). Out of all the significant DEGs, 268 of these genes were shared by the PRP treated macrophages (Figure 3B) whereas only 27 were shared with MSC-CM treated macrophages. Although there were genes associated with catalytic activity (RNASE6, DNMT3L), most of the upregulated genes are commonly associated with inflammatory responses such as CCL22, CCL17, ENSECAG00000031387 or CX3CL1, TIMP3 (Figure 7B). Matching the individual gene profile, the significant pathways included upregulation of inflammatory responses, IL-2 signaling, TNFα and KRAS signaling as well as hypoxia (Figure 7C). Finally, similarly to PRP lysate treatment, ACS also induced a downregulation of MTOR signaling and type 1 interferon signaling, demonstrating some potential to induce favorable changes in the OA joint environment.

### Impact of OT treatment on macrophage polarization

Finally, we evaluated the ability of various OT treatments to affect macrophage polarization; recognizing that polarization is represented by a dynamic state beyond the traditional assessment of M1 vs. M2 (45, 46). Assessment of up- or downregulation of genes associated with M1 versus M2 macrophage phenotypes. These findings are summarized in Supplemental materials 5–7. Gene set lists from GSE5099 specific to *in vitro* derived M1 or M2 human monocyte derived macrophages were used to generate heat maps for genes expressed in the equine macrophages. Macrophages treated with orthobiologics present a mixed phenotype, with both up and down regulation of “M1 up” genes as well as “M1 dn”. Macrophages treated with orthobiologics also showed mixed expression of “leukocyte activation genes” which would be important for modulation of the *in vivo* inflamed joint environment. Looking at a more condensed gene list of commonly known macrophage phenotyping genes (dot plots, Supplemental material 6), for example IFNG is downregulated in IL1b and MSC groups, IL6 is downregulated in PRP and ACS groups. Whereas IL13 and IL4 (M2 gene) is upregulated in PRP and ACS group.

## Discussion

Orthobiologics have been increasingly used in the treatment of equine musculoskeletal disease in recent years, and it is therefore important to understand how these new treatments modulate joint inflammatory responses mechanistically (15, 16, 24, 28). Therefore, the primary goal of this study was to elucidate mechanisms of action of three commonly used biologic therapies on macrophage function and polarization, as synovial macrophages are recognized as one of the most immunologically active cells within the joint and in the progression of OA. Improved understanding of the effects of biologic therapies on key immune effector cells within the joint is fundamental to further designing trials with the correct biomarker endpoints to determine relative biological effects of treatments, including cytokines in synovial fluid or sequencing of synovial fluid or tissue biopsies. Key findings of this study were that all treatments reduced levels of IL-1ß, while MSC-CM induced the greatest increase in anti-inflammatory IL-10 levels, and both PRP lysate and ACS induced lower levels of IL-6 and IP-10 relative to MSC-CM. These findings demonstrate potential beneficial effects of all treatments assessed relative to OA progression, albeit through different mechanisms. RNA sequencing revealed that MSC-CM downregulated inflammatory gene expression while PRP lysate and ACS directed a mixed response with upregulation of both pro- and anti-inflammatory gene pathways and mixed M1/M2 macrophage polarization. This study is the first to report the relative effects of three commonly used orthobiologic formulations in the modification of inflammation induced in macrophages, simulating conditions experienced in inflamed osteoarthritic joints. These findings will inform future studies examining the potential benefits of regenerative therapies *in vivo* in various models of OA in horses.

Multiple cytokines that were differentially secreted by macrophages following treatment with orthobiologics have been implicated to play a role or have prognostic value in assessing the severity of OA. Disruption of the cytokine balance toward a pro-inflammatory state in the pathogenesis of OA has been described to propagate a “vicious cycle” activating catabolic enzymes and contributing to further damage to cartilage, synovium, and intra-articular soft tissue structures (43). IL-1ß has been cited as one of the primary pro-inflammatory cytokines involved in the pathogenesis of OA, among other disease conditions (43), inducing catabolic events including cartilage degradation through mitogen-activated protein kinase (MAPK) signaling, reducing cartilage extracellular matrix *via* ERK activation, and inhibiting collagen synthesis through SOX-9 expression (47, 48). As noted, in this study, IL-1ß secretion was downregulated by all three orthobiologics assessed. Furthermore, concentrations of PGE-2 in synovial fluid have been evaluated to determine degree of joint inflammation as they are consistently elevated in naturally occurring (49) and experimental models of equine OA (50) and lameness in horses in general (51). While PGE-2 was not induced to a significantly greater extent in the IL-1ß stimulated macrophages vs. control, PGE-2 levels were found to be lowest in the MSC and ACS treated groups and were actually significantly upregulated in the PRP lysate group, consistent with the mixed induction of some pro-inflammatory pathways seen with PRP lysate on RNA sequencing analysis.

Interleukin-6 (IL-6) has historically been characterized as pro-inflammatory (52, 53) and documented to be upregulated in joints with osteoarthritis (54, 55), although more recent work has suggested that it may play a more immunomodulatory and not strictly a pro-inflammatory role (56–58). In humans undergoing knee arthroscopy, IL-6 and MCP-1 concentrations have been correlated to higher (worse) intraoperative International Cartilage Repair Society (ICRS) scores, were the greatest predictors of more severe cartilage lesions, and were associated with more prolonged pain postoperatively (59). Both IL-6 and IP-10 were further associated with greater hip OA pain and were detected in both the synovial fluid (IL-6 and IP-10) and synovium (IP-10) of OA vs. healthy patients, indicating distinct inflammatory processes may drive OA in specific joints or at specific time points in disease progression (60). In this study, IL-6 and IP-10 secretion were downregulated by PRP lysate and ACS in comparison to IL-1ß stimulated macrophages. Given the conflicting reports on the relative catabolic vs. pro-chondrogenic effects of IL-6 to equine cartilage, further studies on the global role of IL-6 in equine OA are warranted.

Interleukin-10 (IL-10) is broadly considered to be an anti-inflammatory cytokine through multiple pathways and is primarily synthesized by immune cells and to a lesser extent by chondrocytes within the joint, where it plays a role in cartilage extracellular matrix turnover (61). In this study, MSC-CM maintained levels of IL-10 close to those of the positive control and to significantly higher levels than that induced by treatment with PRP lysate or ACS. In addition, RANTES (regulated upon activation, normal T cell expressed and secreted) has further been associated with recruitment of macrophages and PGE-2 generation following injection in rodent models and reported as a mediator of acute and chronic inflammation (59). In humans, RANTES levels were among the strongest predictors, along with platelet-derived growth factor and vascular endothelial growth factor, of postoperative improvement regardless of initial injury or degree of cartilage degradation at the time of surgery (59). This is interesting in the context that, of the treatments assessed, MSC-CM upregulated RANTES most, resulting in greater secretion of RANTES by macrophages compared to PRP lysate, although no treatment was significantly different from the positive or negative control. These findings shed some light on the relative effects of orthobiologic agents in the context of IL-1ß induced inflammation and highlight the concept that the pathogenesis of OA involves activation of signaling pathways by multiple cytokines (43).

This study represents the first in-depth look at how equine macrophages respond to IL-1ß, a relevant inflammatory cytokine in osteoarthritis progression, quantified *via* transcriptomic analysis. Macrophages were co-cultured with recombinant IL-1ß to model the inflammatory synovial environment that impairs healing and exacerbates OA progression, as IL-1ß has been commonly associated with the osteoarthritic synovial environment as a pro-inflammatory cytokine, in addition to IL-6 and TNF-α (43). In response to IL-1ß stimulus, the ROS pathway, and several key leukocyte activation genes such as TLR1, TLR9, TLR2, IL4, IFNGR1, IL-13 etc. (GO:0045321), were upregulated in IL-1ß treated macrophages compared to the control unstimulated macrophages. Overall, the most upregulated genes were related to inflammatory immune system (SLAMF9, PPBP, TRIB3, GPR84, and DDIT4) and metabolic processes (CHAC1, PSAT1, PEAK3, and CEBPD). Simultaneously, downregulated genes mapped to categories including impaired cell signaling and biological regulation of cellular adhesion and differentiation. Thus, the transcriptomic analysis techniques employed here provided previously unreported insights into how equine macrophages respond to activation by relevant joint inflammatory cytokines, with relevance to the interaction and response of joint cells to orthobiologic treatments in osteoarthritis.

Macrophage polarization states are key in regulation of inflammation in the osteoarthritic joint (62–64). The upregulation of anti-inflammatory genes demonstrated following MSC-CM treatment represents a polarization toward an M2 macrophage phenotype and is consistent with previous reports discussing the importance of MSC-macrophage crosstalk in the maintenance of homeostasis in inflammatory microenvironments and the role of macrophage phenotype switching from M1 to M2 in tissue repair (62, 63) (Supplementary materials 5–7). Published datasets of gene expression include key patterns (65) seen in polarized macrophages *in vitro* that define the transcriptomic response beyond the dichotomy of M1 and M2 (66). While it is recognized that the findings of this study may not be directly compared to human data sets on resting macrophages given species differences and the induced IL-1ß inflammation modeled here, these previous reports provide a baseline from which initial comparisons may be drawn. In this study, the IL-1ß treated macrophages co-cultured with PRP upregulated many genes previously classified as M1 or pro-inflammatory oriented; for example, 41% of the M1 genes found in GSE5099 and several of the “leukocyte activation genes” found in GO:0002269 (leukocyte activation involved in inflammatory response) such as IFN-γ, IL-13, and IL-4, were upregulated, suggesting that PRP lysate polarized macrophages toward an M1 profile in these pathways (Supplementary material 2). Transcriptome abundance, a more sensitive measure than protein levels, further indicated upregulation of TNF-α; of note, these outcomes may lack correlation if protein levels were not high enough to detect *via* multiplex assay or if the stimulus was not sufficient to trigger release but was strong enough to alter transcript abundance. However, overall pathway analysis for PRP lysate-treated samples revealed a global transcriptome that points to a downregulation of the immune response, type I and II interferon pathways, complement and ROS pathways, indicating (as with MSC-CM) the potential utility of PRP lysate to resolve chronic inflammation during OA pathogenesis. Finally, PRP treatment also resulted in the largest number of “uniquely” differentially expressed genes (207 as seen on VENN diagram, Supplementary material 3) of orthobiologic therapies assessed, some of which mapped to angiogenesis, integrin signaling, TGF-ß, and other pathways that produce downstream effects that could potentially contribute to the amelioration of tissue damage and inflammatory cell infiltrate. These results highlight the importance of the non-biased approach used here to analyze transcriptomic response to therapy and investigate the mechanisms of action by which orthobiologic therapies exert an effect.

Treatment of macrophages with ACS also created an apparent shift in the macrophage transcriptome, and, similarly to that seen with PRP lysate treatment, downregulated several inflammatory pathways including ROS, oxidative phosphorylation, and the type II interferon response. The 153 genes unique to the ACS treatment (Figure 3) present sets of genes that, although inflammatory, could be beneficial to recruit innate immune cells to the site of inflammation (toll like receptor (TLR) signaling, Wnt signaling, integrin, glycolysis, DNA replication, and EGF receptor), which may further contribute to the initial phase of tissue repair in traumatic injury. In contrast to MSC-CM, ACS treatment also upregulated several inflammatory pathways (TNF-α, IL-2, Stat 5 signaling, and apoptosis), along with multiple M1 leukocyte activation genes (TNF-α, IFN- γ, IL4, and FOXP1). Similarly to PRP lysate-treated macrophages, approximately 44% of M1-associated genes (GSE5099) were upregulated compared to the 62% M1-associated upregulated genes in the IL-1ß alone group (Supplementary Figure S2). Despite the upregulation of multiple M1 genes, it has been previously reported that the M2 macrophage response encompasses a dynamic spectrum of transcriptomic states ranging from the classic M2 tissue resident macrophage to M2a, b, c and d subtypes depending on which receptor(s) are activated (IL-4, TGF-ß, or glucocorticoids) and their respective downstream effects (67). For example, the M2a phenotype has been found to upregulate peroxisome proliferator-activated receptor (PPAR), STAT6 and STAT3 pathway genes, which were all upregulated in the ACS group. These transcriptomic shifts and plasticity of macrophage polarization further highlight the advantages of using an impartial approach (i.e., bulk RNA sequencing) to investigate cell product derived therapeutics for the treatment of joint diseases.

Caveats to study design include small donor horse sample size, inherent variability in orthobiologic composition between individual donors and products available, and assessment of cytokine concentrations and differential gene expression at a single time point. Different donor horses were used to develop orthobiologic treatments vs. monocyte-derived macrophage cultures simply due to availability at the time the studies were performed. It is further acknowledged that the model employed is not proposed to represent the spectrum of conditions encompassing OA, and likely did not fully capture the chronic inflammatory response seen in longstanding degenerative joint disease nor the potential for orthobiologic agents to exert a longer-term effect in mitigation of disease progression as culture media and macrophages were only assessed at a single time point. The peripheral blood monocyte-derived macrophage model employed is recognized to not fully represent the complexity and spectrum of synovial macrophage phenotypes. In addition, activation of macrophages in this model may have been enhanced through combined stimulation using both IL-1ß and TNF-α, both cytokines found to be elevated in OA, as has been recently described (68). Interpretation of transcriptomic data cannot be used to predict net outcomes in comparing OT treatments but does give us the most in-depth evaluation possible as to what processes are invoked through OT treatment with emphasis on overall pathways, rather than individual genes. Finally, it is acknowledged that substantial variability exists between preparation and composition of orthobiologic therapies, including differences between manufacturers, culture techniques such as serum source and media components for mesenchymal stromal cells, and individual donor factors including the health, time of day and environment of the donor prior to tissue donation (69, 70). For example, the PRP lysate product used (Arthrex ACP) represents a relatively low platelet concentrate with lower levels of platelet derived growth factor (PDGF) and other related growth factors than other commercially available products; it is recognized that other formulations with higher platelet concentrations may have yielded different results. Furthermore, characterization of the PRP lysate and ACS products used here were not supplied, which is recognized as a limitation. Finally, cultures were performed with conditioned media from MSC rather than cells themselves, which was done in an attempt to standardize comparisons between products as much as possible, and it is hypothesized that co-culture with cells would have only accentuated the findings reported. While this study represents an initial comparison of three treatments that are currently commonly employed in equine practice, but it is further acknowledged that multiple others (e.g., alpha-2 macroglobulin, autologous protein solution, adipose derived MSC or MSC-CM, amnion, and urinary bladder matrix) exist, in addition to other formulations of the products investigated here, and that comparison of effect and mechanism of action in future *in vitro* and *in vivo* studies is warranted.

In summary, these findings indicate that commonly used equine orthobiologic therapies exert their actions through various mechanisms including induction of differential cytokine production and gene expression from resident joint tissues that may be beneficial in treatment of osteoarthritis, and further highlight the benefits of employing a non-biased approach to transcriptomic and cytokine analysis to investigate mechanisms of action of these treatments. These studies begin to address a critical gap in our understanding of the relative immunomodulatory properties of regenerative therapies commonly used in equine practice to treat musculoskeletal disease and will serve as a platform from which further *in vivo* comparisons of orthobiologic therapies may build.

## Data availability statement

The datasets presented in this study can be found in online repositories. The names of the repository and accession number(s) can be found below: GEO public genomics data repository, and the accession number is GSE224326.

## Ethics statement

The animal study was reviewed and approved by Colorado State University Institutional Animal Care and Use Committee.

## Author contributions

Study conception and design: LP, LC, SD, LB, LG, and GG. Acquisition of data: LP, LC, LB, RI, and GG. Data analysis and interpretation: GG, LP, LC, SD, LB, and LG. Drafting of manuscript: LP, GG, and LC. All authors contributed to and approved the submitted version of the manuscript.
