# Supplementary figures and images for: Distinct differences in immunological properties of equine orthobiologics revealed by functional and transcriptomic analysis using an activated macrophage readout system
Source: Front Vet Sci. 2023 Feb 16;10:1109473. doi: 10.3389/fvets.2023.1109473 (PMC9978772; doi:10.3389/fvets.2023.1109473)

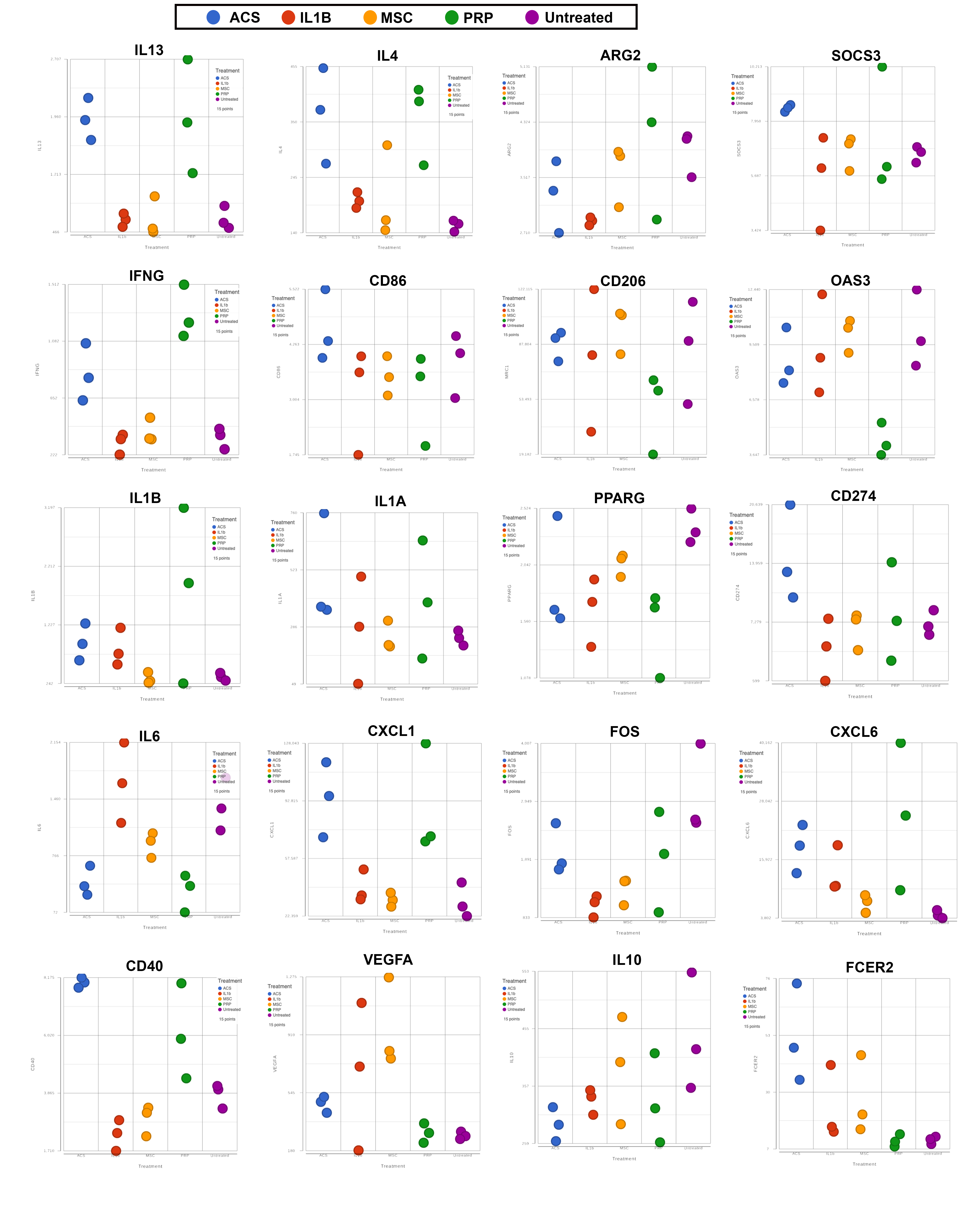

Supplement: Supplementary file 6 [file Image_1.TIF]

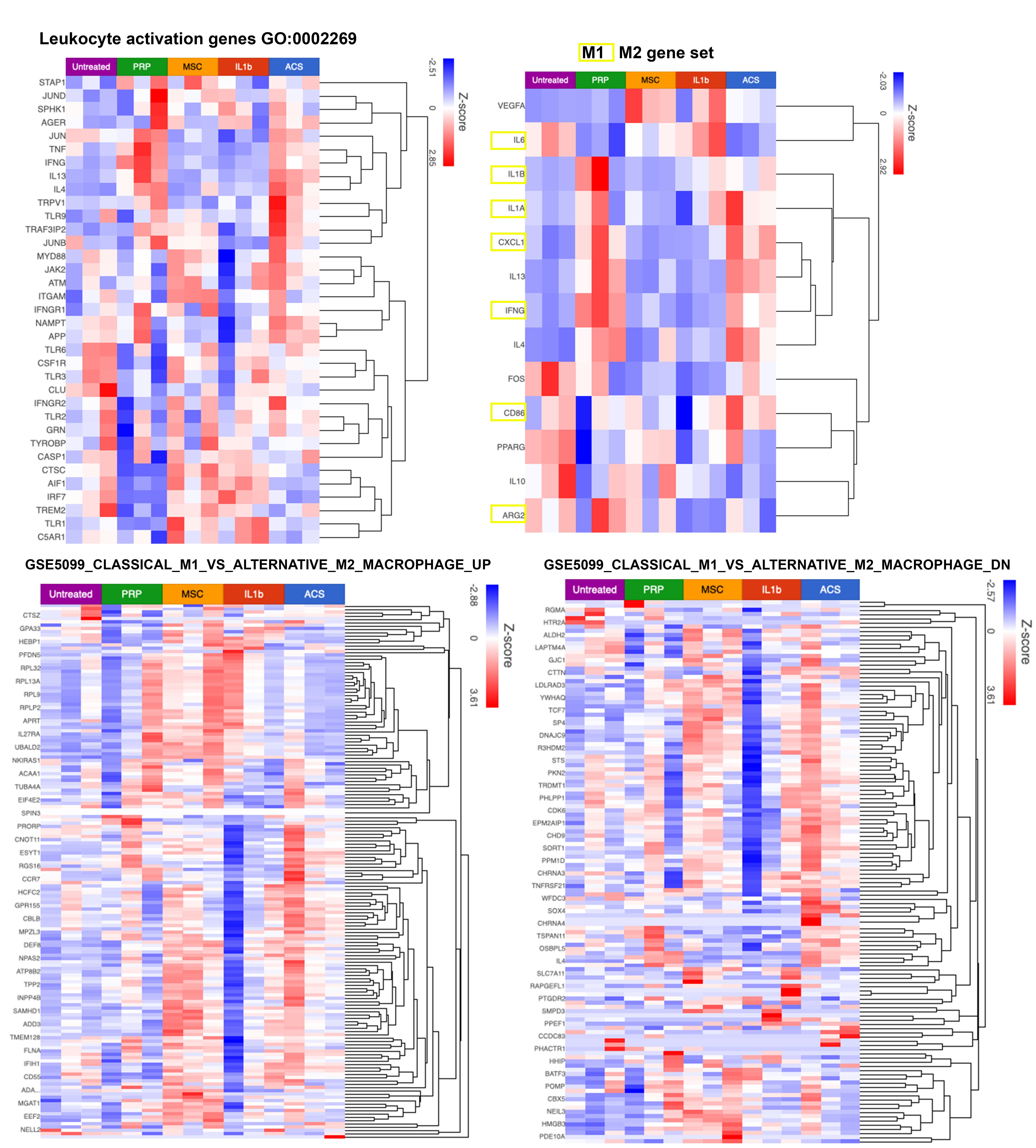

Supplement: Supplementary file 7 [file Image_2.TIF]
